# Recombination and insertion events involving the botulinum neurotoxin complex genes in *Clostridium botulinum *types A, B, E and F and *Clostridium butyricum *type E strains

**DOI:** 10.1186/1741-7007-7-66

**Published:** 2009-10-05

**Authors:** Karen K Hill, Gary Xie, Brian T Foley, Theresa J Smith, Amy C Munk, David Bruce, Leonard A Smith, Thomas S Brettin, John C Detter

**Affiliations:** 1 Bioscience Division, Los Alamos National Laboratory, Los Alamos NM 87545, USA; 2 DOE Joint Genome Institute, Los Alamos National Laboratory, Los Alamos NM 87545, USA; 3 Theoretical Division, Los Alamos National Laboratory, Los Alamos NM 87545, USA; 4 Integrated Toxicology Division, United States Army Medical Institute of Infectious Diseases (USAMRIID), Fort Detrick, MD 21702, USA

## Abstract

**Background:**

*Clostridium botulinum *is a taxonomic designation for at least four diverse species that are defined by the expression of one (monovalent) or two (bivalent) of seven different *C. botulinum neurotoxins *(BoNTs, A-G). The four species have been classified as *C. botulinum *Groups I-IV. The presence of *bont *genes in strains representing the different Groups is probably the result of horizontal transfer of the toxin operons between the species.

**Results:**

Chromosome and plasmid sequences of several *C. botulinum *strains representing A, B, E and F serotypes and a *C. butyricum *type E strain were compared to examine their genomic organization, or synteny, and the location of the botulinum toxin complex genes. These comparisons identified synteny among proteolytic (Group I) strains or nonproteolytic (Group II) strains but not between the two Groups. The *bont *complex genes within the strains examined were not randomly located but found within three regions of the chromosome or in two specific sites within plasmids. A comparison of sequences from a Bf strain revealed homology to the plasmid pCLJ with similar locations for the *bont/bv b *genes but with the *bont/a4 *gene replaced by the *bont/f *gene. An analysis of the toxin cluster genes showed that many recombination events have occurred, including several events within the *ntnh *gene. One such recombination event resulted in the integration of the *bont/a1 *gene into the serotype toxin B *ha *cluster, resulting in a successful lineage commonly associated with food borne botulism outbreaks. In *C. botulinum *type E and *C. butyricum *type E strains the location of the *bont/e *gene cluster appears to be the result of insertion events that split a *rarA*, recombination-associated gene, independently at the same location in both species.

**Conclusion:**

The analysis of the genomic sequences representing different strains reveals the presence of insertion sequence (IS) elements and other transposon-associated proteins such as recombinases that could facilitate the horizontal transfer of the *bonts*; these events, in addition to recombination among the toxin complex genes, have led to the lineages observed today within the neurotoxin-producing clostridia.

## Background

*Clostridium botulinum *is a taxonomic designation for at least four diverse groups of Gram positive spore-forming anaerobic bacteria that produce the most potent naturally occurring toxin known, botulinum neurotoxin (BoNT). Production of BoNT has been the single criterion for inclusion within the *C. botulinum *species and was adopted in order to prevent scientific and medical confusion regarding the intoxication known as botulism. However, this single criterion has resulted in a species designation that encompasses clades of strains that should be considered as four separate species. Phylogenetic analysis of 16S *rrn *genes of *C. botulinum *strains clearly separates them into four Groups (I-IV) and supports this historical classification scheme based upon biochemical and biophysical parameters [1]. Group I contains proteolytic serotype A, B and F strains, as well as bivalent (bv) Ab, Ba, Af, and Bf strains; Group II consists of nonproteolytic (np) and saccharolytic serotype B, E and F strains; Group III consists of serotype C and D strains; and Group IV consists solely of serotype G strains [2]. Group IV has been recognized as a distinct species and its members have been given the additional name of *C. argentinense *[3]. Further Group designations (V and VI) have been proposed for other clostridial species found to express BoNT, such as the BoNT/F-producing *C. baratii *strains and the BoNT/E-producing *C. butyricum *strains [4].

**Figure 1:**
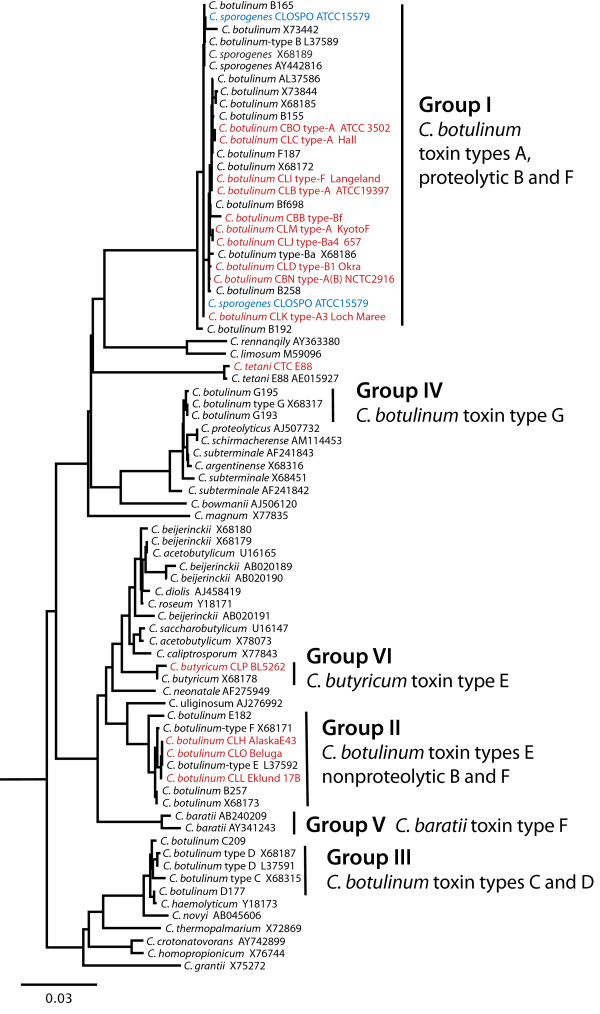
**A 16S *rrn *dendrogram of clostridial species**. The 16S *rrn *genes from the 15 strains examined in this study (13 *C. botulinum *indicated in red, one BoNT/E-producing *C. butyricum *in red and one *C. sporogenes *in blue) were aligned to the 16S *rrn *genes of different *Clostridium *species identified within Genbank via BLAST searches. A maximum likelihood tree using 78 sequences with four outgroup sequences from the *Alkaliphilus *genus (removed) was generated from 1,208 nucleotides. The scale bar of 0.03 represents three point mutations per 100 bases or 3% diversity between sequences. Two 16S *rrn *gene sequences from *C. sporogenes *ATCC 15579 are included. The 16S *rrn *dendrogram illustrates the genetic diversity within the *Clostridium *genus and among strains within the Group I-VI designations.

Figure 1 and previously published 16S *rrn *dendrograms show the relationship of the *bont*-containing strains to each other and to other clostridial species [5,6]. Group I shares a recent common ancestor with nontoxic *C. sporogenes*. Group II is a subset of a more diverse clade that includes other saccharolytic clostridia, such as *C. acetobutylicum*, *C. beijerinckii*, and toxic and nontoxic Group V *C. baratii *and Group VI *C. butyricum*. Group III strains produce BoNT/C, D and mosaic C/D and D/C toxins which share a recent common ancestor with nontoxic *C. novyi*. Group IV, producing BoNT/G, shares a clade with *C. subterminale *and *C. proteolyticus*. Recent microarray analyses of Group I strains confirm the close relationship of the strains with *C. sporogenes *and the disparity in gene content between Groups I and II strains [7].

The 16S *rrn *dendrogram also shows that the tetanus toxin-producing Clostridia, *C. tetani*, occupies a distinct clade when compared to the other clostridial species. This species was one of the first clostridial genomes to be sequenced revealing the presence of the tetanus toxin within a 74 kb plasmid [8]. Recent genomic sequences of different *C. botulinum *strains have revealed single or bivalent *bonts *are located within plasmids as often as within the chromosome [9-11]. Unlike tetanus toxin, which appears uniform from strain to strain, *bont *gene sequence comparisons have identified multiple variants that are recognized as serotypes and subtypes.

Comparisons of the BoNT/A-G protein sequences in strains representing the different Groups show that BoNT protein identities range from 34%-64% among the seven serotypes [9]. In addition, the variation observed in BoNT protein sequences within the serotypes, except in type G, has resulted in designations of BoNT subtypes within a serotype (for example subtypes A1-A5 within BoNT/A).

The discordant phylogeny of the serological classification of the toxins with the 16S *rrn *analyses and Group designations indicates that the *bont *genes have been horizontally transferred between various clostridial lineages. Horizontal gene transfer events are observed within other bacterial species and contribute to bacterial evolution [12]. Although the exact transfer mechanisms active within the clostridia remain unclear, the regions flanking the *bont *and toxin complex genes include partial and complete insertion sequence (IS) elements and gene duplication events indicative of mobile element activity. In addition, the genes of several *bonts *are located within plasmids or phage [9-11]. These findings suggest possible mechanisms that could enable the horizontal transfer of *bont *[13]. Recombination events within the *bont *genes (mosaic *bont/c/d *and *bont/a1/a3 *for example) and within the *ntnh *gene that precedes the *bont *gene have been observed and contribute significantly to BoNT diversity [5,6,13,14]. Although the three plasmids that contain *bont/a3*, *bont/a4, bont/bv b *or *bont/b1 *genes are largely homologous, each shows regions of inversions and deletions [9].

Because the toxin complex genes appear to move among the clostridia, they cannot be used to infer the phylogenetic relationships of the host bacteria. However, the sequences and the locations of the *bont *gene clusters provide clues to earlier gene transfer and recombination events. In order to better understand these events, we compared the available genomic sequences of several strains within the Group I, II and VI designations. Chromosome and plasmid synteny were analysed and the specific locations and sequences flanking the *bont *complex genes were examined within *C. botulinum *types A, B, E and F strains and a *C. butyricum *type E strain. Plasmid locations for the *bont/np b *gene within the Eklund 17 B strain and for the *bont/bv b *and *bont/f *genes within the bivalent Bf strain were identified. A detailed examination of the toxin complex genes and their flanking regions revealed recombination and insertion events that have contributed to the diversity observed today.

## Results

### Chromosomal and plasmid synteny

The chromosomal and plasmid sequences from strains representing multiple *C. botulinum *serotypes and subtypes of A, B, E and F, two bivalent strains (BoNT/Ba4, BoNT/Bf), a BoNT/E-expressing *C. butyricum*, a *C. tetani *and a *C. sporogenes *strain (Table 1) were compared in order to investigate their overall organization or synteny. Comparisons of the completed chromosomal sequences of the three BoNT/A1 strains (ATCC 3502, ATCC 19397, Hall) revealed that these strains are nearly identical in genomic organization (data not shown). The history of the three strains is not clear, however, they appear to be different strains isolated from foodborne outbreaks of botulism [15]. The serotype A Hall strain is distinctive in that it produces a high concentration of toxin in culture [16]. Unique to the ATCC 3502 strain is the presence of a 16 kb plasmid [17]. Neither this intact plasmid nor its plasmid sequences were found within the chromosomes of the other two BoNT/A1 strains.

**Table 1:** List of analyzed genomes.

Species	Subtype^1^	Strain	Group	Locus tag ID^2^	Genbank accession^3^	Toxin complex	BoNT location^5^
*C. botulinum*	A1	ATCC 3502	I	CBO	AM412317/AM412318	HA-A1	chr/oppA
*C. botulinum*	A1	ATCC 19397	I	CLB	CP000726	HA-A1	chr/oppA
*C. botulinum*	A1	Hall	I	CLC	CP000727	HA-A1	chr/opp
*C. botulinum*	A1(B)	NCTC 2916	I	CBN	ABDO02000001-49	orfX-A1, HA-(B)	chr/arsC, chr/oppA
*C. botulinum*	A2	Kyoto-F	I	CLM	CP001581	orfX-A2	chr/arsC
*C. botulinum*	A3	Loch Maree	I	CLK	CP000962/CP000963	orfX-A3	plasmid
*C. botulinum*	Ba4	Strain 657	I	CLJ	CP001083/CP001081/CP001082	orfX-A4, HA-bvB	plasmid
*C. botulinum*	B1	Okra	I	CLD	CP000939/CP000940	HA-B1	plasmid
*C. botulinum*	Bf	-----	I	CBB	ABDP01000001-70	HA-bvB, orfX-F	plasmid
*C. botulinum*	prot F	Langeland	I	CLI	CP000728/CP000729	orfX-F	chr/arsC
	
*C. botulinum*	npB	Eklund 17B	II	CLL	CP001056/CP001057	HA-npB	plasmid^6^
*C. botulinum*	E1	Beluga	II	CLO	ACSC01000001-16	orfX-E1	chr/rarA
*C. botulinum*	E3	Alaska E43	II	CLH	CP001078	orfX-E3	chr/rarA
*C. butyricum*	E4	BL 5262	II	CLP	ACOM01000001-13	orfX-E4	chr/rarA
	
*C. tetani*	tetanus	E88	-	CTC	NC 004557/NC 004565	p21-NT	Plasmid
	
*C. sporogenes*	N/A	ATCC 15579	I	CLOSPO	ABKW02000001-4	N/A	N/A

^1^Subtype designations as listed in Hill et al, 2007

^2^Locus tag ID designations listed in GenBank and Hill et al, 2007

^3^Accessions listed as chromosome/plasmid

^4^orfX = orfX1, orfX2, orfX3; HA = HA17, HA33, HA70 accessory proteins

^5^Location: chr = chromosome, oppA = oppA/brnQ operon, arsC = arsC operon, rarA = rarA operon; plasmid = plasmid

^6^Plasmid does not share homology with Group I plasmids

**Figure 2:**
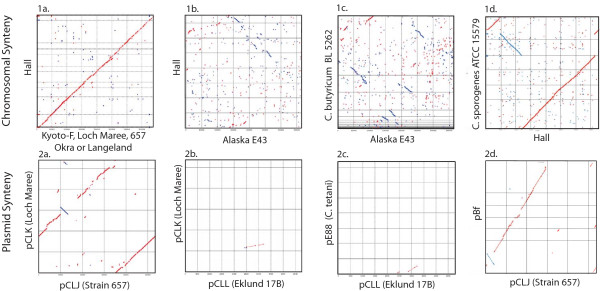
**Chromosomal and plasmid synteny plots**. Panels 1a-d or 2 a-d show four synteny plots of either chromosomal or plasmid sequence alignments, respectively. The reference sequence listed on the *x*-axis was queried with the strain sequence listed on the *y*-axis. The red dots indicate forward matches of the sequence comparisons: the blue dots indicate reverse compliment matches. The continuous diagonal line in the plot in panel 1a illustrates the overall chromosomal organization or synteny shared between the proteolytic strains of Hall and either the Kyoto-F, Loch Maree, 657, Okra or Langeland strains. Panel 1b and 1c plots compare Hall and *C. butyricum *BL 5262 to the BoNT/E-producing Alaska E43 strain, where little synteny is observed. In panel 1d four contigs of *C. sporogenes *ATCC 15579 are compared to the Hall strain and reveal genomic synteny and a 701 kb inversion between the two species. Panels 2a-d examine plasmid synteny. The diagonal lines in panel 2a illustrate that the Loch Maree pCLK has a similar organization to pCLJ with a small 16.7 kb inversion that includes the *bont/a3 *relative to the *bont/a4*. Panels 2b and 2c show that pCLL within Eklund 17B does not share synteny either to pCLK or pE88 that contains the tetanus toxin. In panel 2d four contigs of the Bf strain show synteny to pCLJ and the 16.7 kb inversion of *bont/a4 *relative to the *bont/f*.

Figure 2 (panel 1a) compares the genomic synteny of the Hall BoNT/A1 strain to other *C. botulinum *Group I strains representing serotypes A, B and F. The plot shows that the chromosomes of strains representing four BoNT/A subtypes (BoNT/A1-A4), BoNT/B1 or BoNT/F share similar organization. In contrast, there is little chromosomal synteny between the Group II *C. botulinum *serotype E strains and the Group I Hall strain or the *C. butyricum *type E strain (Figure 2, panel 1b, 1c). The two BoNT/E-producing *C. botulinum *strains (Alaska E43 and Beluga) were similar to each other and also to the npBoNT/B Eklund 17B strain (data not shown). These comparisons revealed a large (404 kb) inversion within the Eklund 17B chromosome relative to the *C. botulinum *serotype E strains that is not in a region containing the *bont/e *gene cluster. No chromosomal synteny was observed when the *C. botulinum *Group I and Group II strain sequences were compared to the *C. tetani *E88 strain (data not shown). A comparison of the four contigs of *C. sporogenes *ATCC 15579 to the Hall BoNT/A1 strain (Figure 2, panel 1d) revealed genomic synteny and a large 701 kb inversion between the two species. The four panels (1a-d) contrast the genomic organization among Group I, II and VI strains and show that Group I strains share a similar gross chromosomal organization to each other and to *C. sporogenes*, which differs from Group II and VI strains.

Plasmid synteny was also examined by comparing the *bont*-containing plasmids (pCLK with *bont/a3*, pCLJ with *bont/a4 *and *bont/bv b*, pCLD with *bont/b1*) from Group I to each other and to the Group II pCLL with *bont/np b *and pE88 in *C. tetani*. These plasmids each contain genes encoding: 329 proteins (pCLK); 195 proteins (pCLD); 305 proteins (pCLJ); 54 proteins (pCLL); and 59 proteins (pE88). Although the plasmids containing *bont/a3*, *bont/a4 *and *bont/b1 *vary in size (148 kb - 270 kb), Figure 2 panel 2a shows large regions of conserved organization among these plasmids and a small inversion (16.7 kb) that contains the *bont/a3 *relative to the *bont/a4*.

The genomic sequence of the Group II B strain, Eklund 17B, revealed the location of the *bont/np b *within a small (47.6 kb) plasmid, pCLL, that was unique when compared to other *bont*-containing plasmids. Synteny plots show that pCLL differs from pCLK (Figure 2 panel 2b) and pE88, the plasmid within *C. tetani *that contains tetanus toxin (Figure 2 panel 2c). None of the *C. botulinum *plasmids (pCLK, pCLJ or pCLD) shared synteny to *C. tetani *pE88 (data not shown).

Although the sequence data for the Bf strain is incomplete, four Bf contigs share synteny to the bivalent pCLJ that contains *bont/a4 *and *bont/bv b *(Figure 2 panel 2d). The same inversion (16.7 kb) identified in panel 2a is observed when the contigs are compared to pCLJ. The evidence for the plasmid location of *bont/bv b *and *bont/f *is supported by the sequence homology of the four contigs to pCLJ and a detailed examination of the location of the *bont/bv b *and *bont/f *is described later.

These results show that the Group I *C. botulinum *A, B and F strains share a similar chromosome organization to each other and to *C. sporogenes *but not to the Group II nonproteolytic B strain or serotype E strains, the Group VI BoNT/E-producing *C. butyricum *or *C. tetani*. The plasmids containing *bont/a3*, *bont/a4*, *bont/bv b*, *bont/b1 *or *bont/f *gene clusters also show similarity to each other but not to the *C. tetani *pE88 or pCLL containing *bont/np b*. Comparisons between the Group II *C. botulinum *BoNT/E or npBoNT/B-producing strains revealed that their chromosomal backgrounds share synteny with each other but not with the Group VI *C. butyricum *type E strain. These relationships confirm the different genomic backgrounds within *C. botulinum *and *C. tetani *and support the 16S *rrn *analyses and historical *C. botulinum *Group designations.

### Components of the BoNT gene clusters

**Figure 3:**
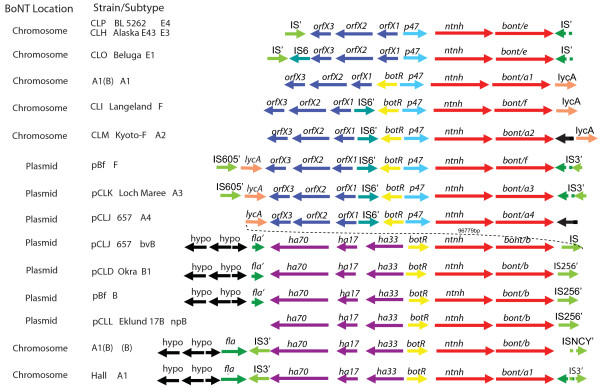
**BoNT complex and flanking regions in different strains**. The *bont *gene cluster, flanking regions and location (chromosome or plasmid) are indicated for the different strains. The *orfX *cluster (*orfX3-orfX2-orfX1-(botR)-p47-ntnh-bont *complex genes) is present in the BoNT/E-producing strains (*C. botulinum *and *C. butyricum*), the BoNT/A1 of the A1(B) strain, serotype F (BoNT/F and BoNT/bvF) and the BoNT/A2-A4 subtypes. The *ha *cluster (*ha70-ha17-ha33-botR-ntnh-bont *complex genes) is present in the serotype B strains containing BoNT/bvB, BoNT/B1, npBoNT/B, BoNT/(B) and BoNT/A1 of the Hall strain. The flanking regions consist of IS elements, flagellin (fla), *lycA *and hypothetical (hypo) proteins. The prime symbol indicates a partial gene.

The arrangement and composition of the toxin gene clusters in strains representing the different serotypes and subtypes of *C. botulinum *and BoNT/E-producing *C. butyricum *are shown in Figure 3. A comparison of these regions shows, in general, that the BoNT gene is located in either of two conserved toxin gene cluster arrangements, composed of either the *ha70-ha17-ha33-botR-ntnh-bont *complex genes (abbreviated *ha *cluster) or the *orfX3-orfX2-orfX1-(botR)- p47-ntnh-bont *complex genes (abbreviated *orfX *cluster). The characteristics of the different proteins and their arrangements have been previously reported for strains representing the different serotypes [5,6]. The toxin complex proteins, with the exception of the regulatory protein BotR (P21), are thought to provide a protective role for the BoNT in the gastrointestinal tract [18]. There is evidence that the hemaagglutinin (HA) proteins may also help facilitate the absorption of BoNT from the intestines into the bloodstream [19]. While all of the genes within the *ha *cluster express proteins that are part of the toxin complex, the expression and function of the orfX proteins within the *orfX *cluster remain unknown. The presence of genes that encode the complex proteins in each of the different serotypes suggests that these proteins must play a role in expression, stability and/or transport of the BoNT.

Figure 3 shows that the *ha *gene cluster is found within serotype A subtype BoNT/A1 strains and all of the serotype B strains, including the gene cluster harboring the silent *bont/(b) *gene within BoNT/A1(B) strains. The *orfX *gene cluster is found within all of the other strains examined here, including BoNT/A2, BoNT/A3, BoNT/A4 strains and the *bont/a1 *gene cluster within the BoNT/A1(B) strain. It is also found within the proteolytic BoNT/F Langeland strain, the *bont/f *gene cluster in the bivalent Bf strain, the BoNT/E1 and BoNT/E3 strains and the BoNT/E-producing *C. butyricum *strain.

The *bont/a1 *gene appears to be the only *bont *so far identified within either of the two types of toxin complexes. The *bont/a1 *gene in strains ATCC 3502, ATCC 19397 and Hall is located within the *ha *cluster and the *bont/a1 *within the BoNT/A1(B) strain, as well as several other BoNT/A1 strains, is located within the *orfX *cluster [20]. It appears that the location of the *bont/a1 *gene within the *ha *cluster resulted from a recombination event in the middle of the serotype B *ntnh *gene that has been previously reported [21]. The first half of the *ntnh *gene in the BoNT/A1 strain is 99.7% identical to the *ntnh *within serotype B strains. After a recombination event occurring at approximately 1,965 nucleotides from the start codon of the 3,594 bp gene, the second half of the *ntnh *gene is equally similar to the *ntnh *gene in serotypes A2, A3 and A4 (90 to 95% identity) strains. This event has resulted in a *bont/a1 *gene residing within an *ha *cluster that contains a hybrid or recombinant B/A *ntnh *gene.

The *ntnh *recombination event locating the *bont/a1 *gene within the *ha *cluster has resulted in a very successful lineage that is frequently identified in botulism cases. The many strains representing this event, such as ATCC 3502, ATCC 19397 and Hall, contribute to the acceptance of the *ha *cluster in association with the *bont/a1 *gene. However, the *orfX *cluster is more likely to be the ancestral toxin gene cluster containing the *bont/a1 *gene, as indicated by the location of the other *bont/a *subtype genes (*bont/a2 bont/a3 *and *bont/a4*) and the *bont/a1 *gene of the silent B strains within the *orfX *cluster. In addition, the *bont/a1 *genes within the *ha *cluster are located in a different region of the chromosome from the *bont/a1 *genes in the *orfX *cluster, as described below.

### Location of the BoNTs within the chromosome

**Figure 4:**
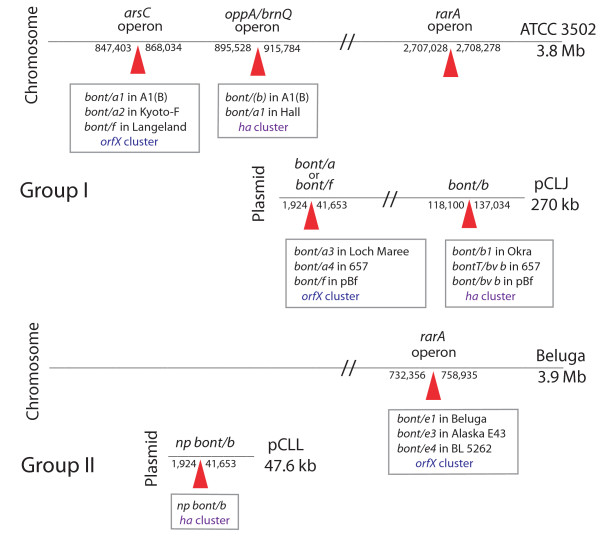
**Relative locations of the different *bonts *within the chromosome or plasmid**. Three operons (designated *arsC*, *oppA/brnQ *and *rarA*) within the chromosome of the ATCC 3502 or Beluga strain show where the various *bonts *are located within the different strains. The *bont/a1 *of the A1(B) strain, *bont/a2 *of the Kyoto-F and the *bont/f *of the Langeland strain are located within the *arsC *operon. The *bont/(b) *within the A1(B) strain and the *bont/a1 *within the Hall strain is located within the *oppA/brnQ *operon. The *rarA *operon contains the *bont/e *complex within the Beluga, Alaska E43 or *C. butyricum *BL 5262 strains. The relative locations of the *bonts *in the Group I plasmids are indicated in pCLJ. One site contains either the *bont/a3 *in the Loch Maree strain, the *bont/a4 *in the bivalent 657 or the *bont/f *in the Bf strain. Another site contains either the *bont/b1 *in the Okra strain, the *bont/bv b *in 657 or *bont/bv b *in the Bf strain. The *bont/np b *location within pCLL within the Eklund 17B strain is indicated. This figure shows the common sites of the *bonts *in different strains providing evidence that the *bonts *are not randomly located within the chromosome or plasmid.

Because the strains within each *C. botulinum *Group showed genomic synteny when compared to each other, the chromosomal or plasmid location of each *bont *gene was examined to determine if the regions containing the different *bont *genes had similar features. This analysis revealed that the *bont *genes in these strains are not randomly distributed but rather are found within three specific sites within the chromosome: (1) the *arsC *operon that contains either the *bont/a2, bont/f *or the *orfX-bont/a1 *of the silent BoNT/A(B) strains; (2) the *oppA/brnQ *operon that contains either the *ntnh-*recombinant (*ha*) *bont/a1 *or *bont/(b); *and (3) the *rarA *operon which contains the *bont/e *within the *C. botulinum *and *C. butyricum *type E strains. Figure 4 shows the location of these sites in relation to the ATCC 3502 or Beluga chromosome: the *arsC *operon at approximately 847 kb, the *oppA/brnQ *operon at approximately 895 kb and the *rarA *operon at approximately 2,704 kb.

The *arsC *gene is part of a group of genes (*arsA*, *arsB*, *arsC*, *arsD*, and *arsR*) that encodes for proteins involved in arsenic reduction. BoNT/A1, BoNT/A1(B), BoNT/A2, and BoNT/F strains contain all five genes, but BoNT/A3, BoNT/Ba4 and BoNT/B1 strains lack genes for *arsA*, *arsB *and *arsD*. Recently, it has been shown that certain BoNT/B2 strains lacking the full gene complement are sensitive to arsenic, while BoNT/B2 strains containing all five genes are relatively resistant to arsenic [22].

**Figure 5:**
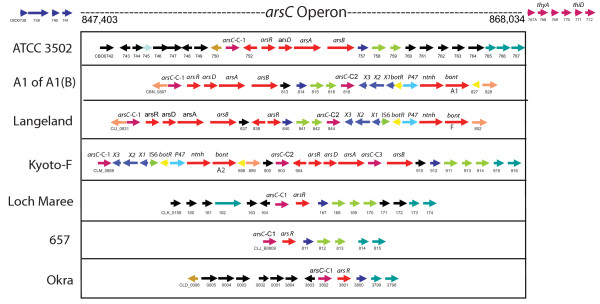
**Comparison of the *arsC *operon in different strains**. The region of the *arsC *operon within the ATCC 3502 strain was compared to the *arsC *region in other strains. The horizontal arrows indicate coding sequences (CDSs). Gene designations are labelled above the arrow. GenBank locus IDs are labelled below the arrow. The first CDS was given the full GenBank locus ID followed by an abbreviated ID that uses only the last 2-3 digits. At this site (847 kb - 868 kb) there is no toxin gene cluster within ATCC 3502; however, this site contains the *bont/a1 *of the A1(B) strain and the *bont/f *within the Langeland strain. The components in this region are depicted in the Kyoto-F, Loch Maree, 657 and Okra strains. The regions flanking the *arsC *operon are similar upstream and downstream in each of these strains.

An expanded view of the *arsC *operon in Figure 5 shows the different constituents within this location in the different strains. Within this approximately 20 kb region three *bont *genes can be found: the orfX-*bont/a1 *of BoNT/A(B) strains; the proteolytic *bont/f; *and the *bont/a2*. A striking similarity is seen between the region surrounding the *bont/a1 *cluster and that surrounding the *bont/f *cluster. These two different serotypes contain many of the same genes in the same order in this location. The *bont/a2 *gene cluster is also located here, but this region is not as similar to the region within the BoNT/A1 or BoNT/F strains as they are to each other. As has previously been reported, the *bont/a2 *is located in between two copies of the *arsC *[23]. Other strains, such as those containing *bont/a3*, *bont/a4*, *bont/bv b *or *bont/b1 *genes, have no *bont *genes within this region.

**Figure 6:**
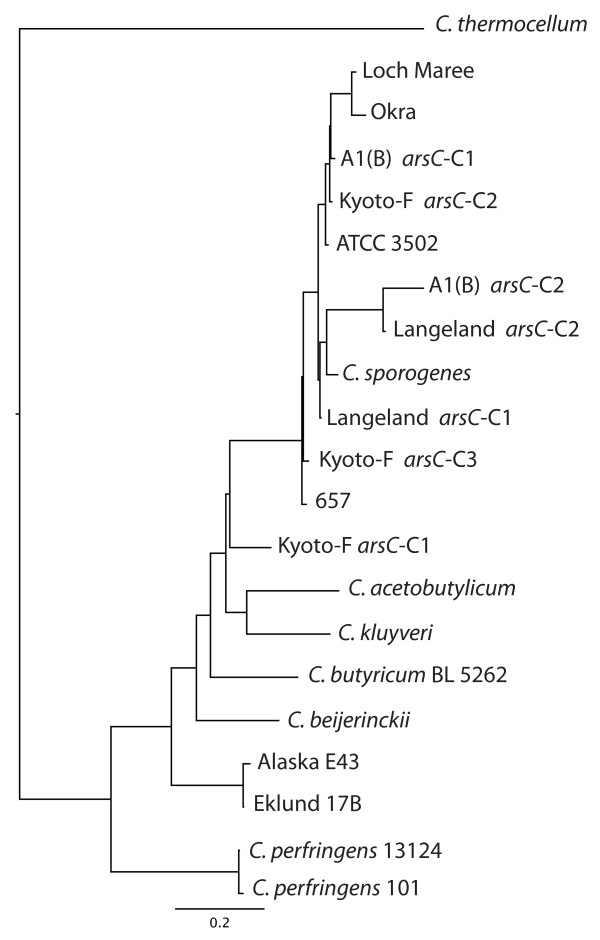
**Dendrogram of *arsC *gene**. The 392 nucleotides of *arsC *(arenate reductase) were compared among *C. botulinum *strains and other clostridial species. Where multiple copies of the *arsC *were present within a strain, the copies are designated as C-1, C-2 or C-3 based upon their location within the operon shown in Figure 5. The dendrogram illustrates that the *arsC *copies within the same strain are different from each other and that the *arsC *sequences from Groups I, II and VI strains differ.

Since some of these strains contain multiple *arsC *genes, a dendrogram of the various copies was created to compare genes within and among the strains (Figure 6). The *arsC *dendrogram shows that the sequences of the *arsC *genes are not identical within a strain or between the strains. It also shows that the three copies within the BoNT/A2 strain differ from each other, as do the two copies found within BoNT/A1(B) and BoNT/F strain. The single *arsC *gene within *C. sporogenes *is more closely related to one of the copies within the Group I strains. The copy within the Eklund 17B and Alaska E43 strains are nearly identical but differ from the *arsC *within *C. butyricum*.

**Figure 7:**
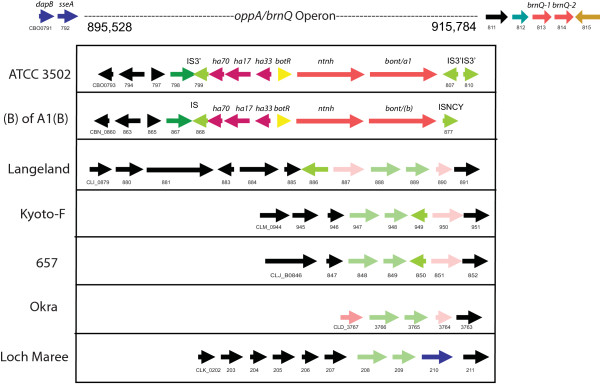
**Comparison of the *oppA/brnQ *operon in different strains**. The region of the *oppA/brnQ *operon within the ATCC 3502 strain was compared in the different strains. The horizontal arrows indicate coding sequences (CDSs). Gene designations are labelled above the arrow. GenBank locus IDs are labelled below the arrow. The first CDS was given the full GenBank locus ID followed by an abbreviated ID that uses only the last two to three digits. In this region (895 - 915 kb) the *bont/a1 *within the ATCC 3502 strain and the *bont/(b) *of the A1(B) strain are located. No *bont *genes within the other strains of Langeland, Kyoto-F, 657, Okra and Loch Maree are located here. The regions flanking the *oppA/brnQ *operon are similar upstream and downstream in each of these strains.

About 25 kb downstream from the *arsC *operon in the ATCC 3502 strain is the *oppA/brnQ *operon where the *bont/(b) *gene, or the *ha *cluster BoNT/A strains, are located (Figure 7). This site is named for the *oppA*, extracellular solute binding protein, and *brnQ*, branched chain amino acid transport protein, located here. This is the only site where a *bont/(b) *gene, although silent due to a mutation, was identified within the chromosome; the *bont/b1 *and *bont/bv b *genes in strains analyzed as part of this study were located within plasmids. This site does not contain the *bont *genes in the BoNT/A2, BoNT/A3, BoNT/A4 or BoNT/B1-producing strains. The *oppA/brnQ *operon was not present within the serotype E strains, the BoNT/E-producing *C. butyricum*, or the npBoNT/B strain.

**Figure 8:**
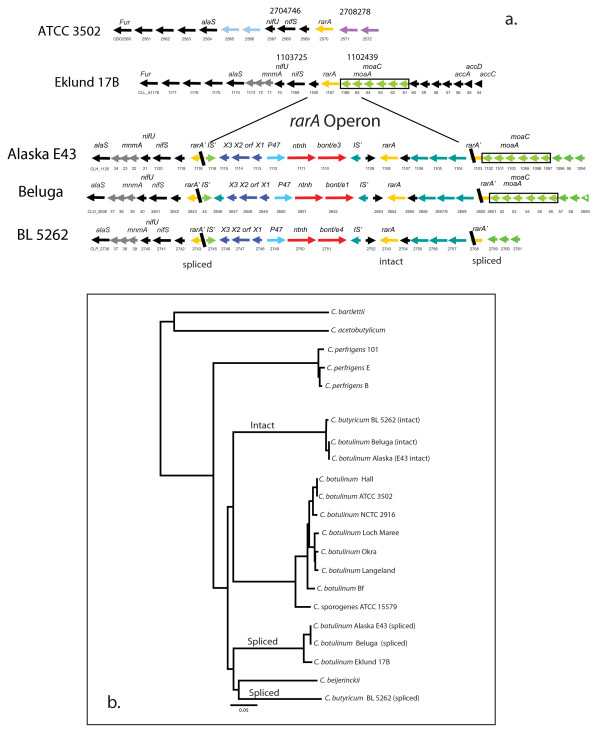
**(a) Location of the *RarA *operon within *C. botulinum *and *C. butyricum *strains and (b) dendrogram of *rarA *genes from different clostridial species**. The *rarA *operon in the ATCC 3502 strain is compared to the *rarA *operon in the Group II and VI strains. In the Eklund 17B strain the *rarA *gene is intact. However, in the Alaska 43, Beluga and *C. butyricum *BL 5262 strains, the *rarA *gene is split and a *bont/e *gene cluster has been inserted. Note the similarity of the components within the inserted sequence and that it also contains an intact *rarA *gene. The regions flanking the *rarA *operon are similar upstream and downstream in the Group II strains. (b) The dendrogram of *rarA *genes shows that some strains contain two copies of *rarA*, one that is intact and one that is split from the insertion of the *bont/e *complex genes. The 1,195 nucleotides of *rarA *from both intact and split genes were compared; the sequences of split *rarA *genes were spliced together to make full-length genes. The dendrogram shows that the sequences of the spliced *rarA *in *C. botulinum *Alaska E43 and Beluga type E strains are similar to each other but are different from the spliced *rarA *in *C. butyricum *BL 5262. This difference indicates that the insertion of the toxin gene cluster occurred as two separate events in each species. The inserted/intact *rarA *sequences in both of these species are similar indicating a common source.

At approximately 2704 kb within the ATCC 3502 chromosome (1.102 Mb in Eklund 17B) is the location of the *rarA *operon. No *bont *genes are located here in the Group I proteolytic strains. However, in the BoNT/E-producing *C. botulinum *(Beluga and Alaska E43) and *C. butyricum *(BL 5262) strains, the *rarA *gene is split and the *bont/e *gene cluster and other genes are inserted. Figure 8(a) shows the similarity of the *rarA *region in the npBoNT/B strain and the two BoNT/E-producing species (*C. botulinum *and *C. butyricum*) and also the gene organization of the inserted sequence. Although these regions appear similar, the *bonts *in the strains are in different locations - the *bont/np b *is located within a small plasmid whereas the *bont/e *genes are located within the chromosome.

The gene sequence of the split *rarA *in the serotype E strains can be spliced together to encode an intact fully functional protein. The location of the split (codon 102) is in the same site in both the *C. botulinum *and *C. butyricum *strains. Interestingly, the inserted sequences not only contain the *bont/e *gene cluster, but also contain another *rarA *gene that is intact. Therefore, these strains retain an intact copy of *rarA *in addition to the one that is split.

Figure 8(b) compares the nucleotide sequences of the spliced and intact *rarA *gene in these strains and other species. The dendrogram shows that the intact (inserted) *rarA *are almost identical to each other in the BoNT/E-producing *C. botulinum *and *C. butyricum *strains, suggesting a common source. However, the sequences of the spliced *rarA *genes within these *C. botulinum *and *C. butyricum *strains are not identical. The spliced *rarA *within the Beluga and Alaska E43 strains are almost identical to each other and very similar to the Eklund 17B strain *rarA *sequence. The different sequences of the *rarA *genes that are split by the *bont/e *insertion in *C. botulinum *and *C. butyricum *show that these were separate events occurring in different bacterial backgrounds.

The mechanism of the insertion event likely involves the *rarA *protein, which is a resolvase involved in recombination or insertion events of transposons. Transposon activities within Gram positive bacteria are not well characterized but are known to be responsible for genetic exchange of antibiotic resistance genes and/or genomic islands in other bacteria such as *Staphylococcus aureus *methicillin resistance, for example [24]. The *rarA *insertion site was likely targeted by the presence of a *rarA *gene within the inserted region. The presence of an IS element and a transposon resolvase involved in horizontal gene transfer suggests that either or both could have played a role in the insertion of the *bont/e *gene cluster into the chromosome.

### Location of the BoNTs within plasmids

The plasmid location of the *bont/a3*, *bont/a4*, *bont/bv b *and *bont/b1 *genes from the analysed strains has been previously described [9,10]. The *bont/np b *gene was recently identified by pulsed field gel electrophoresis to be located within a small plasmid [11]. The genomic sequence data for the Eklund 17B strain verified the presence of *bont/np b *within a unique 47.6 kb plasmid. In addition, the location of the *bont/bv b *and *bont/f *within a plasmid (pBf) in the Bf strain was identified based upon synteny results and the high sequence homology of four Bf strain contigs (ABDP01000018.1, ABDP01000023.1, ABDP01000034.1 and ABDP01000069.1) with pCLJ, pCLD and pCLK. The comparisons of pCLJ to the Bf contig sequences yielded the following results: 99% identity, 89% coverage to contig ABDP01000023.1 (68.4 kb) that contains *bont/f*; 99% identity, 81% coverage to contig ABDP01000018.1 (84.3 kb) that contains *bont/bv b; *96% identity, 52% coverage to contig ABDP01000034.1 (16.8 kb); and 98% identity, 65% coverage to contig ABDP01000069.1 (0.8 kb).

**Figure 9:**
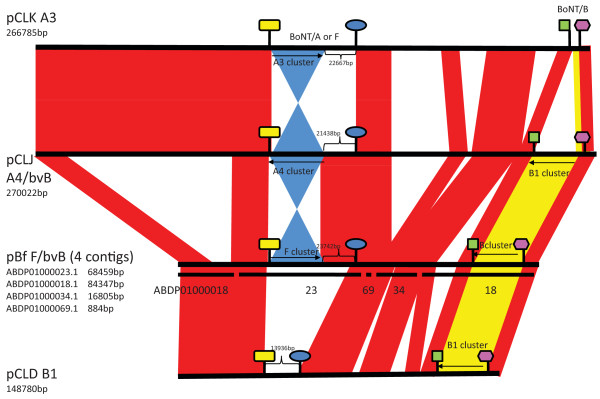
**Plasmid synteny among pCLK, pCLJ, pCLD and pBf**. Three fully sequenced plasmids (pCLK, pCLJ and pCLD) are compared to four contigs of the Bf strain that showed identity to pCLJ by BLASTN analysis. Regions of homology among the bivalent pCLJ, pCLK, pCLD and four pBf contigs is indicated in red and the toxin regions containing of *bont/a3*, *bont/a4 *or *bont/f *are coloured in blue or the *bont/bv b *and *bont/b1 *in yellow. The comparisons show the similar location of the *bont/bv b *and *bont/b1*among the 3 plasmids. The *bont/f *and *bont/a3 *also have similar locations but are inverted in relation to *bont/a4*. The four Bf contigs include ABDP01000018.1 (84.3 kb), ABDP01000023.1 (68.4 kb), ABDP01000034.1 (16.8 kb), ABDP01000069.1 (0.8 kb) and were ordered according to pCLJ. The coloured symbols are expanded in Figure 10 to detail the genes located in these regions.

These results are depicted in Figure 9 where the sequences of the four plasmids, bivalent pCLJ, pCLK, pCLD and the four pBf contigs, are compared. Regions of homology among these plasmids are indicated in red and the toxin regions of *bont/a3*, *bont/a4*, *bont/b1, bont/bv b *and *bont/f *are indicated in yellow or blue. The figure cannot accurately depict pBf because the sequence data is incomplete (170 kb), however, it does appear that the *bont/f *and *bont/bv b *are located within a plasmid that is very similar to the bivalent pCLJ. It is interesting to note the similar locations of the *bonts *in the two plasmids, where it appears the *bont/a4 *is replaced with *bont/f*.

**Figure 10:**
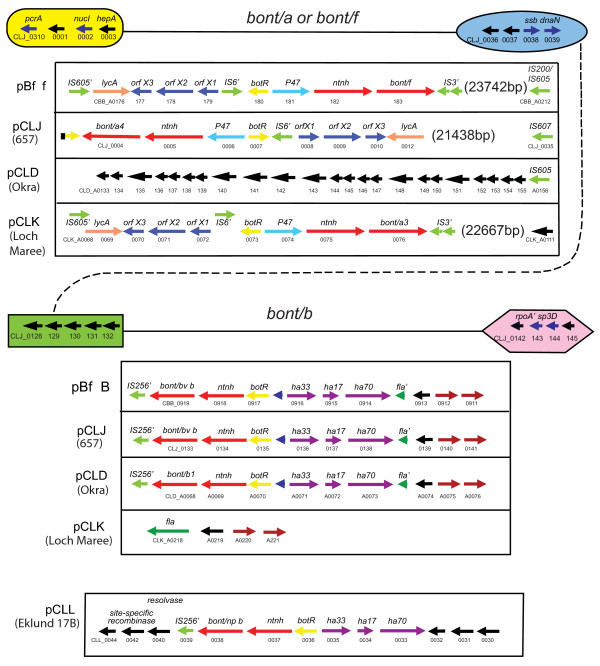
**Plasmid regions containing *bonts***. This is an expanded image of the regions between the symbols in Figure 9 and provides details of the genes located within the different plasmids in these areas. The horizontal arrows indicate coding sequences (CDSs). Gene designations are labelled above the arrow. GenBank locus IDs are labelled below the arrow. The first CDS was given the full GenBank locus ID followed by an abbreviated ID that uses only the last two to three digits. The figure shows that the *bonts *within the plasmids in these Group I strains are located in either of two sites. One location (between the yellow and blue symbols) contains either the *bont/f *of the Bf strain, the *bont/a4 *of the 657 strain or the *bont/a3 *of the Loch Maree strain. The numbers in parentheses, such as 23,742 bp in the pBf panel, indicate additional sequence in that region that is not detailed but is shown in Figure 9. The other plasmid site that contains the *bont/b *in several plasmids is depicted between the green and purple symbols. This region contains the *bont/bv b *or *bont/b1 *in the Bf strain, 657 strain or the Okra strain. The bottom panel depicts the region containing the *bont/np b *within the Eklund 17B strain. This region shares no similarity to the Group I plasmids.

An examination of the *bont *locations within the plasmids shows that, as with the chromosome, there are specific sites within the plasmid where the *bonts *are located (Figure 4, 10). The two plasmid sites contain either: (1) the *bont/a3 *gene cluster, the *bont/f *gene cluster from the Bf strain or the *bont/a4 *gene cluster from the 657 strain; or (2) the *bont/b1 *gene cluster, the *bont/bv b *gene cluster from the 657 strain or the *bont/bv b *gene cluster from the Bf strain. Interestingly, the location of *bont/a1 *or *bont/f *genes at the same site within the plasmid was also observed within the chromosome.

The second plasmid site within the Group I strains contains either the *bont/bv b *or the *bont/b1 *gene. However, the *bont/np b *is located within a very different plasmid and host background from the proteolytic strains. Examination of the regions flanking the *bont/np b *reveals that downstream is an IS element, a transposon-associated resolvase and site-specific recombinase. Like *bont/e*, the *bont/np b *is another example where a *bont *is in proximity to a transposon-associated protein involved in recombination and insertion events within a Group II background.

### Recombination within the *ntnh *gene

**Figure 11:**
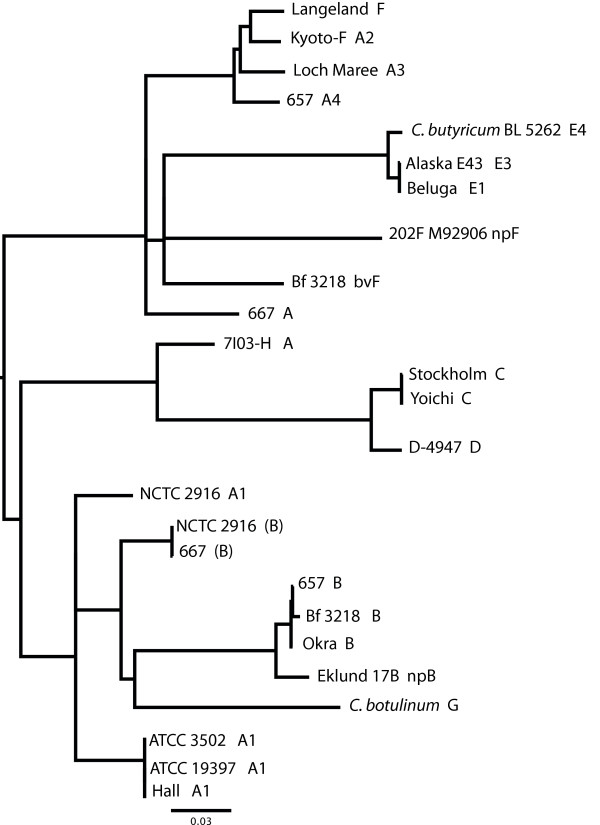
**Dendrogram of the *ntnh *gene in different BoNT-producing strains**. The dendrogram of 3,471 nucleotides of the *ntnh *gene shows the variation within this gene. Some of the *ntnh *sequence variation in the strains is due to recombination events. The location of the *ntnh *in ATCC 3502, ATCC 19397 and Hall strain close to the *ntnh *within the serotype B strains resulted from a recombination event midway within the *ntnh *gene resulting in a recombinant B/A *ntnh*, that is a partial B *ntnh *and partial A *ntnh*. Another similar recombination event in *ntnh *of the 7I03-H strain has resulted in a recombinant C/A *ntnh*, that is a partial C *ntnh *and a partial A *ntnh*. The Langeland F *ntnh *location in the dendrogram near the serotype A strains of Kyoto-F, Loch Maree and 657 resulted from a recombination event near the 3' end or following the *ntnh *gene where a *bont/f *was inserted. The dendrogram illustrates the variation in the *ntnh *genes from multiple serotypes and the location of recombinant *ntnh *genes.

The *ntnh *gene has been consistently located within the toxin complexes in strains representing each of the seven serotypes (A-G) and has been identified as a region of recombination among strains of different serotypes [21]. The *ntnh *dendrogram (Figure 11) illustrates the variation observed among the different serotypes. The *ntnh *within the A2-A4 subtypes (*orfX *cluster) is very different from the A1 subtypes (*ha *cluster) represented by the ATCC 3502 or the A1(B) strains. A recombination event has occurred approximately midway within the *ntnh gene *between a serotype B *ntnh *and a serotype A *ntnh *resulting in a hybrid or recombinant B/A *ntnh*; this recombination event has placed the *bont/a1 *within the *ha *cluster usually associated with *bont/b*.

Another recombination event was observed in the BoNT/A2-producing 7I03-H strain associated with an infant botulism case in Japan, evident from its location within the dendrogram. The first 2000 bases in this recombinant *ntnh *are almost identical with a BoNT/C1 *ntnh *(99.6% identity) and the final 1582 bases are 99.1% identical to the *ntnh *of the BoNT/A2 Kyoto-F strain (designated C/A *ntnh*) [25]. The site of this recombination event is in the same region, but not in the same site, as the hybrid B/A *ntnh *described above.

The dendrogram also illustrates that the *ntnh *gene of the BoNT/A2-4 subtypes and the serotype F Langeland strain are very similar to each other, yet their *bonts *differ. A comparison of the *ntnh *genes for BoNT/A2 Kyoto-F and BoNT/F Langland shows them to be 97.0% identical for the first 3,443 nucleotides, but the identity decreases to 51.0% in the final 58 nucleotides. This finding indicates that a possible recombination event has occurred either in the 3' terminus of the *ntnh *gene and/or in the intergenic region between the *ntnh *and *bont *genes. The occurrence of this recombination event is also supported by the location of the serotype F Langeland *ntnh *gene with the *ntnh *genes of BoNT/A2,/A3, and/A4 strains in the dendrogram, and not with the *ntnh *genes of other serotype F strains.

These examples show the ability of the *ntnh *gene from the toxin complex of serotypes A and C, B and A and the 3' terminus or the intergenic region between an A *ntnh *and the *bont/f *genes to recombine; such recombination events have contributed to the variation observed. These events also illustrate the proximity of bacteria containing these genes to each other within an anaerobic environment that allows exchange and recombination.

## Discussion

Comparisons of the complete and shotgun sequence data from strains representing the Group I and II strains of *C. botulinum *and a *C. butyricum *type E strain were performed in order to further understand the variation observed among the BoNT-producing clostridia and to examine the unusual attributes observed within the species. These include the presence of similar *bonts *in different genomic backgrounds (*bont/e *in *C. botulinum *and *C. butyricum *for example), the presence of different *bonts *in similar backgrounds (serotype A proteolytic B and F *C. botulinum *strains) and the existence of bivalent strains. New technologies have made genomic sequencing more affordable and rapidly provide a wealth of sequence information that molecularly describes an organism. This study utilized the clostridial genomic sequence data and generated comparisons of: the 16S *rrn *genes from various clostridial species; the genomic synteny among strains; the locations of *bont *toxin clusters; and the components in their flanking regions. The data ties previous historical research with molecular results and increases our understanding of the species.

The molecular data supports the historical species Group I-IV classification system for *C. botulinum *based upon biochemical and physical properties. Comparisons of the organization of the genomic sequences in synteny plots presented here confirm that serotype A, B, and F of proteolytic Group I strains share a similar *C. sporogenes *genetic background. Likewise, the genomic organization within the Group II nonproteolytic strains that express B, E and F toxins share similarity to each other. The 16S *rrn *dendrogram shows that the different Groups I-IV within the *C. botulinum *species designation are clearly as distinct as other clades of clostridia that have been classified or named as separate species.

The location of the *bont *gene in these strains revealed that the sites are not randomly distributed in the host genomes. The *bont *and associated cluster genes are located within plasmids of varying sizes (47.6 - 270 kb) as well as within the chromosome. Franciosa *et al*. recently examined the location of the toxin cluster in 63 BoNT/B-producing *C. botulinum *strains using pulsed field gel electrophoresis; they discovered that each of the toxin gene clusters were located within plasmids ranging in size from ~55 to ~245 kb [11].

The presence of the toxin cluster within either plasmids, or within the chromosome in strains of the same or different serotypes, is consistent with horizontal transfer events mediated by plasmids or phage and recombination events mediated by mobile genetic elements such as transposons. These events result in the integration of the *bont *genes into different locations (plasmids, chromosome) and different host backgrounds (Group I-VI), as is observed within the BoNT-producing clostridia. The detailed examination of the *bont *locations reveals that these events occur with a greater frequency by homologous or targeted transposition rather than random or novel integration events.

The species also appears to undergo active recombination within the toxin complex genes, particularly at multiple sites within the *ntnh *gene. Examples of recombination include: (1) *ntnh - *the hybrid B/A *ntnh *placing the *bont/a1 *within the *ha *cluster of serotype B and the hybrid C/A *ntnh *placing the *bont/a2 *following a C/A *ntnh *hybrid; (2) *bont - *the hybrid *bont/a2 *gene consisting of *bont/a1 *and *bont/a3*; *bont/c/d *and *bont/d/c *hybrids; and (3) *ntnh/bont - *the site between the *ntnh *and *bont *genes placing a *bont/f *following a *bont/a2 ntnh *gene. These recombination events compound the confusion of the taxonomy of the species and make it difficult to clearly describe the strains with the current nomenclature. Clearly, from the examples listed above, the multiple recombination events have significantly contributed to the genetic diversity observed in the *bonts*.

This study provides the first molecular information to explain the unusual observation of a *bont/e *within both *C. botulinum *and *C. butyricum *type E strains. By examining the *bont/e *location within the two species, an insertion event was identified which targeted the same *rarA *gene. The *rarA *is a transposon-associated gene with recombinase activity that could explain the precise excision and integration of the *bont/e *in the two species. Interestingly, the comparison of sequences of the spliced and intact *rarA *genes revealed that this insertion event occurred separately in the two species, yet the inserted region containing the *bont/e *gene was from a common source.

Other transposon-associated proteins were identified downstream from the *bont/np b *where an IS element, resolvase and site-specific recombinase are located. Unfortunately, Gram positive transposons are not well characterized and elude detection because they lack perfect inverted repeats flanking the transposed region or are not replicated in the process [26]. Although specific transposons were not identified near the toxin complex genes, transposon-associated proteins were found. The identification of these proteins, the presence of the toxin complex in different host backgrounds, its location within the chromosome as often as within plasmids and the identification of specific targeted insertion sites in the same or different species implicate transposon activity as at least one mechanism for *bont *movement.

The genomic analyses also discovered the location of two *bont *genes within plasmids, the *bont/np b *in the Eklund 17B strain and the *bont/bv b *and *bont/f *within the Bf strain. The *bont/np b*-containing plasmid could have been horizontally transferred to a Group II bacterial background, or it could have been the result of a transposon-mediated insertion into a unique plasmid. Likewise the *bont/bv b *and *bont/f *location within a plasmid homologous to the bivalent pCLJ with the *bont/a4 *replaced with *bont/f *shows that the two sequenced bivalent strains contain *bonts *in similar locations and that the *bonts *are distant to each other. It is interesting that, within the two sequenced bivalent strains, the *bonts *are within either an *ha *cluster or an *orfX *cluster. These different clusters could provide differing protection or expression of the *bont*.

The finding that the *ntnh *gene has recombined to place the *bont/a1 *within the *ha *cluster associated with BoNT/B strains helps resolve the perception of the 'normal' toxin cluster associated with *bont/a1 *strains. The success of the *ha *toxin cluster strains, as evidenced by their widespread isolation in conjunction with human botulism cases, indicates that the *ha *components must confer some cultural or toxicity advantage that is not yet clearly understood.

## Conclusion

This study, which compares 15 clostridial genomic sequences, was undertaken in order to identify the underlying events that result in the genetic diversity within the *C. botulinum *species. As more genomic sequences become available, additional clues to understanding this complex species and its many toxin types and subtypes will be uncovered. This molecular analysis provided: (1) a 16S *rrn *dendrogram of the clostridial species that included recently sequenced members; (2) synteny plots that visualize chromosomal and plasmid gene organization; (3) the identification of common locations of the *bonts *within the chromosome and plasmid; (4) the components of the *bont*-containing regions that identify common features: (5) a description of an insertion event mediated by a transposon-associated resolvase placing *bont/e *in both *C. botulinum *and *C. butyricum *type E strains; (6) plasmid analyses which show that the *bonts *within the Bf strain and npBoNT/B strain are located within a plasmid; and (7) the identification and examples of recombination within the *ntnh *gene, *bont *gene and the region between these two genes.

The findings illustrate that the *bonts *within the clostridia insert, recombine and are exchanged both within a species and among species. The presence of *bont *genes within stable plasmids that are not lost suggests the genes confer some survival advantage to the host bacteria. Whether the *bont *gene is within a plasmid or chromosome, a single or bivalent arrangement or within the *orfX *or *ha *toxin gene cluster, the toxin has been both retained in, and spread among, a variety of different clostridial species termed Groups. The toxin complex genes have undergone recombination, insertion and horizontal gene transfer events that have yielded many variations of the *bont *gene, thereby producing the toxin serotypes and subtypes. Horizontal gene transfer events and genomic rearrangements are important mechanisms for bacterial survival and evolution. Within the clostridia these attributes have enabled the *bont *genes to continue to survive in different clostridial host backgrounds and environments.

## Methods

### Strains

Table 1 lists the strains examined in this study. They represent *C. botulinum *A, B, E and F serotypes and subtypes, including two bivalent strains (BoNT/Ba4, BoNT/Bf), a strain containing both *bont/a1 *and *bont/b *gene clusters where the *bont/b *gene is not expressed (BoNT A1(B)) and a BoNT/E4-expressing *C. butyricum*; a *C. tetani *and a *C. sporogenes *strain was included for comparison [8]. Some genomic sequences were complete or in several large contigs and others were whole genome shotgun sequences.

### Genomic annotation

Annotation of the assembled genome sequence was carried out with the genome annotation system GenDB [27] and RAST server [28]. A combined gene prediction strategy was applied by means of the GLIMMER 2.0 system and the CRITICA program suite [29] along with postprocessing by the RBSfinder tool [30]. tRNA genes were identified with tRNAscan-SE [31]. The deduced proteins were functionally characterized by automated searches in public databases, including SWISS-PROT and TrEMBL [32], Pfam [33], TIGRFAM [34], InterPro [35], and KEGG [36]. Additionally, SignalP [37], helix-turn-helix [38] and TMHMM [39] were applied. Finally, each gene was functionally classified by assigning clusters of orthologous groups (COG) number and corresponding COG category [40] and gene ontology numbers [41].

### Genome and plasmid comparisons

Homology searches were conducted at the nucleotide and amino acid sequence level using BLAST [42]. In order to obtain a list of orthologs from bacteroidete genomes, a Perl script that determines bidirectional best hits was written; for example, genes g and h were considered orthologs if h was the best BLASTP hit for g and vice versa. E values of 10^-15 ^were acceptable. A gene was considered strain specific if it had no hits with an E value of 10^-5 ^or less. Additional genomic comparisons and dotplot analyses were performed with genome alignment tools, such as MUMmer2 [43], NUCmer [44] and the web interface Artemis Comparison Tool (ACT) [45].

The comparison of toxin gene island insertion patterns was identified using the ACT alignment program at the default settings. Predicted toxin gene island insertion sites were identified from sequence alignments and breakpoint sites were further manually curated. Gene definition was manually annotated by inspecting BLASTP results and sequence alignments. The gene name and locus ID were assigned based on the NCBI Reference Sequence file. Insertion sequence (IS) elements were identified and classified by using the IS Finder database [46].

Plasmid analysis of the Bf contigs was performed by using BLASTN with the pCLJ sequence and 70 Bf contigs. All sequences scoring above the E value cutoff at 1e-20 were extracted for further comparison using the PROmer program from MUMmer package. Four putative pBf sequences from contigs ABDP01000018.1, ABDP01000023.1, ABDP01000034.1 and ABDP01000069.1 were aligned to pCLJ sequences. MUMmerplot was used to display the four contigs (pBf) that were ordered according to pCLJ reference coordinates.

### Dendrograms

DNA alignments were created with a combination of Sequencer software , PAUP , MUSCLE , CLUSTAL-W  and hand editing with BioEdit  software and were gap stripped then analysed using PHYLIP  with dnadist with the F84 model of evolution and a transition to transversion ratio of 2.0 (default) and neighbor joining algorithms. Dendrograms were rendered with FigTree . Intra- and inter-serotype BoNT gene recombination was explored with SimPlot [47] and BioEdit.

## Abbreviations

BoNT: botulinum neurotoxin; bv: bivalent; npBoNT/B: nonproteolytic botulinum neurotoxin B; HA: hemaagglutin; ACT: Aremis Comparison Tool; IS: insertion sequence.

## Authors' contributions

TS and LS initiated the DNA sequencing of some of the strains, assisted in drafting figures and data interpretation; AM, DB, TB and CD were instrumental in the genomic sequencing of some of the strains; BF created dendrograms and contributed to interpretation and writing; GX performed sequence analysis, drafting of figures and data interpretation; KH drafted the manuscript and figures and assisted in data interpretation.
